# Uremic mouse model to study vascular calcification and “inflamm-aging”

**DOI:** 10.1007/s00109-022-02234-y

**Published:** 2022-08-02

**Authors:** Markus Tölle, Cornelia Henkel, Jaqueline Herrmann, Christoph Daniel, Milen Babic, Mengdi Xia, Anna M. Schulz, Kerstin Amann, Markus van der Giet, Mirjam Schuchardt

**Affiliations:** 1 Department of Nephrology and Medical Intensive Care, Charité-Universitätsmedizin Berlin, Cooperate member of Freie Universität Berlin and Humboldt Universität zu Berlin, Campus Benjamin Franklin, Hindenburgdamm 30, 12203 Berlin, Germany; 2 Department of Nephropathology, Friedrich-Alexander-Universität Erlangen-Nürnberg, Krankenhausstraße 8-10, 91054 Erlangen, Germany; 3 Second Clinical Medical Institution of North, Department of Nephrology, Sichuan Medical College (Nanchong Central Hospital), Sichuan Province, Nanchong, 63700 China

**Keywords:** Calcification, Cardiovascular, Chronic inflammation, Chronic renal insufficiency, Inflammation, Vascular calcification

## Abstract

Calcification and chronic inflammation of the vascular wall is a high-risk factor for cardiovascular mortality, especially in patients with chronic uremia. For the reduction or prevention of rapid disease progression, no specific treatment options are currently available. This study aimed to evaluate an adenine-based uremic mouse model for studying medial vessel calcification and senescence-associated secretory phenotype (SASP) changes of aortic tissue to unravel molecular pathogenesis and provide a model for therapy testing. The dietary adenine administration induced a stable and similar degree of chronic uremia in DBA2/N mice with an increase of uremia blood markers such as blood urea nitrogen, calcium, creatinine, alkaline phosphatase, and parathyroid hormone. Also, renal fibrosis and crystal deposits were detected upon adenine feeding. The uremic condition is related to a moderate to severe medial vessel calcification and subsequent elastin disorganization. In addition, expression of osteogenic markers as Bmp-2 and its transcription factor Sox-9 as well as p21 as senescence marker were increased in uremic mice compared to controls. Pro-inflammatory uremic proteins such as serum amyloid A, interleukin (Il)-1β, and Il-6 increased. This novel model of chronic uremia provides a simple method for investigation of signaling pathways in vascular inflammation and calcification and therefore offers an experimental basis for the development of potential therapeutic intervention studies.

**Graphical abstract:**

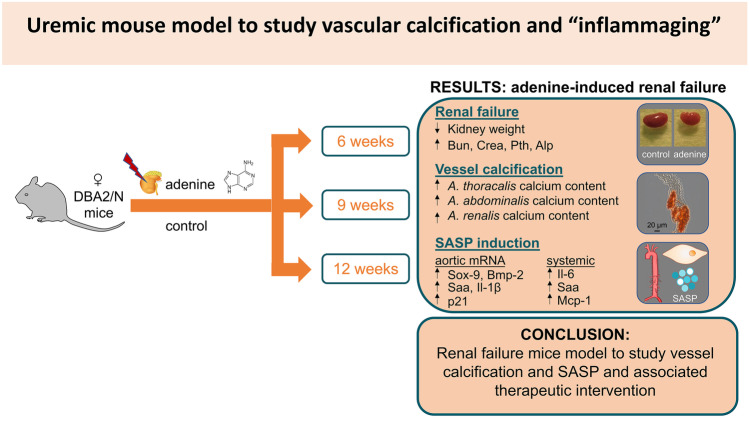

**Supplementary Information:**

The online version contains supplementary material available at 10.1007/s00109-022-02234-y.

## Introduction

Vascular disease is a major cause of morbidity and mortality, especially in patients of higher age or with chronic kidney disease (CKD) [1–3]. Medial arterial calcification (MAC) of the vessel wall is a prominent pathology in CKD patients with rapid progression in later renal disease stages and with a significant association between CKD and cardiovascular mortality [1, 3]. MAC is associated with progressive deposits of hydroxyapatite crystals in the medial layer of the vessel wall. This results in rheological and hemodynamic changes with an increase in pulse wave velocity, left ventricular hypertrophy, and heart failure [3]. Until now, no specific treatment options exist for reduction or prevention of disease progression [1, 3].

Various factors are associated with the formation of MAC and are also increased under CKD condition including levels of serum inorganic phosphate (Pi), calcium (Ca), alkaline phosphatase (Alp), and parathyroid hormone (Pth) [3]. In addition, a transformation of vascular smooth muscle cell (VSMC) reminiscent of osteogenesis is closely associated with the pathogenesis of MAC [2]. This is followed by upregulation of ossification markers, e.g., bone morphogenetic protein 2 (Bmp-2) and SRY box transcription factor 9 (Sox-9) [3]. Although the initial drivers of MAC pathogenesis are not fully elucidated [3], recent studies verified that besides osteogenic transformation, molecular pathways of cellular senescence and inflammation are involved [3, 4]. Accumulating evidence indicates a causal link of cellular senescence and subsequent senescence-associated secretory phenotype (SASP) switch of VSMC in the vessel wall [5]. This leads to chronic disease with tissue dysfunction and chronic inflammation [5]. Various cellular stressors may induce senescence [5], e.g., inhibition of the cell cycle by p21 [6]. Pro-inflammatory cytokines such as interleukin (Il)-1β, Il-6, and serum amyloid A (Saa) are known to be part of the cellular SASP and secreted by vascular cells, e.g., VSMC [7]. For Il-1β, it is already known that it can promote osteogenic differentiation and induction of VSMC calcification [8, 9].

To study signaling pathways for MAC, various models have been developed to investigate MAC: in vitro cell culture models, ex vivo settings on aortic rings, and in vivo rodent CKD models [10, 11]. For studying the complex pathogenesis of MAC, in vivo rodent CKD models are typically necessary. Surgical and non-surgical rodent models exist for CKD induction that also differ in administered diet components [11]. Both the severity of CKD and associated mortality as well as the severity of MAC vary between the protocols [11]. The non-surgical CKD induction by adenine diet becomes a suitable alternative to achieve a reproducible and stable CKD in rats and mice with good tolerability [11–13]. As adenine has a low solubility, it precipitates in renal tubules leading to tubular dilation, renal necrosis, and fibrosis [11]. Several studies have already investigated CKD induction in mice using adenine-enriched diet with protocol differences in diet ingredient concentration, time of induction, and primary read-out of the study [13–20].

Our study aims to develop an adenine-based model of uremia induction in female DBA2/N mice for studying MAC and SASP shift in the vessel wall reproducing the human clinical course of CKD-associated MAC. This model intended to contribute to unravel disease pathogenesis and test novel therapeutic options for lowing the high cardiovascular morbidity and mortality of CKD patients.

## Materials and methods

### Animal study design

Female DBA2/N mice (*n* = 52) were purchased from Charles River at the age of 5–6 weeks. The mice were kept on a 12-h light/dark cycle and a room temperature of around 22 °C with chow and water ad libitum. Normal and experimental chow was purchased from Ssniff (Soest). Mice were divided randomly into 3 cohorts (diet duration: 6, 9, and 12 weeks) with 2 groups per cohort. One group was fed an adenine-enriched diet containing 0.2% adenine, 6% proteins, 1% calcium, and 1% phosphate, whereas the mice of the other group were fed a comparable diet without adenine (Figure S1). A detailed list of diet ingredients and their proximate contents is given in the Supplementary Material (Tables S1 and S2).

Health status of the animals was checked daily; the body weight was monitored once per week for the first 2 weeks, and from week 3 on two to three times per week during the trial period. At the end of each trial period, animals were sacrificed by intraperitoneal injection of pentobarbital (400 mg/kg body weight). Blood was taken from each animal in heparinized tubes. Plasma samples were collected via centrifugation. Plasma samples were aliquoted and stored at − 80 °C until measurement. Organ specimens were prepared for cryo- and formalin-fixation.

### Plasma chemistry

Blood urea nitrogen (Bun), Ca, Pi, Alp, and creatinine (Crea) were analyzed using the blood dry chemistry analyzer (Fuji). Pth1-84 (TECO Medical Group) and Saa (ICL) were quantified via enzyme-linked immunosorbent assay (ELISA) according to the manufacturer’s recommendations. The cytokine plasma concentration was quantified using the Bio-Plex system (BioRad, software version 6.1) with the Milliplex™ Cytokine Kit (Millipore) according to the manufacturer’s recommendations.

### Calcium content

Extracellular Ca content of different vascular beds was performed as previously published [21, 22].

### Histological staining

Paraffin-embedded tissue of the kidney and aorta was cut in 4-µm serial sections. Deparaffinized sections were subsequently stained using hematoxylin/eosin (H&E) protocol modified according to Mayer, Alizarin Red (pH 4) staining, Perjodate Acid Schiff (PAS), elastic tissue fibers Verhoeff’s van Gieson (EVG), Masson’s trichrome stain, and von Kossa staining using standard protocols. Images were acquired using a Zeiss AxioVert 200 light microscope with ZEN 2 software (Zeiss, blue edition) or a BX60 upright microscope equipped with a CX30 camera and cellSense software (all from Olympus Germany GmbH). Representative images were taken at 100–400 × magnification.

### mRNA gene expression

Cryo-conserved aortic tissue was homogenized using the Tissue Ruptor with disposable probes (Qiagen). Ribonucleic acid (RNA) was isolated using Trizol® (Fisher Scientific) according to the manufacturer’s instructions. The RNA was reverse transcribed using the High-Capacity Complementary Deoxyribonucleic Acid (cDNA) Reverse Transcription Kit™ (Applied Biosystems). For the quantitative determination of messenger ribonucleic acid (mRNA) expression, the iQ™ SYBR Green supermix with the CFX384 real-time polymerase chain reaction (PCR) detection system (Biorad, CFX software version 3.1) was used. Each sample was performed as a technical duplicate for real-time PCR. Normalization for each sample was done using ß-actin, ribosomal protein 13 a (Rpl13a), and peptidylprolyl isomerase A (Ppia) as housekeeper genes. The oligonucleotide sequences are given in Table S3.

### Statistical analysis

Statistical analysis was performed by using GraphPad Prism software (version 8). Data are presented as mean ± standard error of mean (SEM). To compare means of relevant groups, Mann–Whitney *U* test was used, with *p* < 0.05 considered statistically significant.

## Results

### Animal health status

The diet was well tolerated from all mice with an overall mortality of 0% during the study period. As expected, the control mice gained weight over the trial period. The adenine diet caused an overall progressive weight loss over the observation period of 12 weeks. During the first 2 weeks, the adenine-treated mice lost weight, while afterwards barely gained weight over the trial period. The initial and final body weights per group are given in Table 1. The time-dependent weight differences for all groups are given in Figure S2.

**Table 1:** Initial and final body weight per group

Experimental group	Animal number	Initial body weight (g)	Final body weight (g)
**Control** (6 weeks)	6	19.07 ± 0.57	20.48 ± 0.53
**Adenine** (6 weeks)	9	18.68 ± 0.26	16.37 ± 0.50*
**Control** (9 weeks)	6	19.28 ± 0.30	22.22 ± 0.62
**Adenine** (9 weeks)	11	19.65 ± 0.38	17.53 ± 0.56*
**Control** (12 weeks)	6	19.78 ± 0.48	22.82 ± 0.45
**Adenine** (12 weeks)	14	19.41 ± 0.32	15.94 ± 0.37*

The weight process is given in Figure S2. Mean ± SEM. ^*^p < 0.05 adenine vs. respective control

### Clinical parameters of renal failure

As expected from previous studies, the adenine diet induces time-dependent renal failure in mice, detectable via change in laboratory chemistry parameters and histological changes in renal structures [13, 15, 16].

The adenine diet induced a time-dependent onset in renal disease in mice with accompanying changes in blood parameters (Table 2). Compared to controls, Bun and Ca were significantly increased after 6 and 12 weeks and Crea, Pth (1–84), and Alp after 6, 9, and 12 weeks. Interestingly, the plasma concentration of Pi did not differ significantly between the groups (Table 2).

**Table 2:** Plasma biochemistry

**Experimental group**	**Bun** (mmol/L)	**Crea** (mmol/L**)**	**Pth** (pg/mL)	**Ca** (mmol/L)	**Pi** (mmol/L)	**Alp** (U/L)
**Control** (6 weeks)	2.45 ± 0.30	0.13 ± 0.02	345.1 ± 137.0	2.23 ± 0.07	2.95 ± 0.24	171.3 ± 16.73
**Adenine** (6 weeks)	5.08 ± 0.58*	0.39 ± 0.06*	997.3 ± 125.9*	2.71 ± 0.07*	3.40 ± 0.27	228.9 ± 8.45*
**Control** (9 weeks)	9.30 ± 2.23	0.24 ± 0.07	653.4 ± 164.6	2.37 ± 0.16	2.54 ± 0.40	150.2 ± 9.87
**Adenine** (9 weeks)	14.40 ± 2.36	0.41 ± 0.06*	2,430 ± 389.9*	2.45 ± 0.12	3.27 ± 0.36	257.2 ± 24.14*
**Control** (12 weeks)	8.54 ± 1.44	0.17 ± 0.02	608.5 ± 185.1	2.13 ± 0.07	3.96 ± 0.33	124.0 ± 5.75
**Adenine** (12 weeks)	41.11 ± 6.19*	0.54 ± 0.03*	4,380 ± 522.0*	2.61 ± 0.10*	4.80 ± 0.46	241.6 ± 19.94*

Mean ± SEM. ^*^p < 0.05 adenine vs. respective control

*Bun* blood urea nitrogen, *Crea* creatinine, *Pth* parathyroid hormone, *Ca* calcium, *Pi* inorganic phosphate, *Alp* alkaline phosphatase

**Fig. 1:**
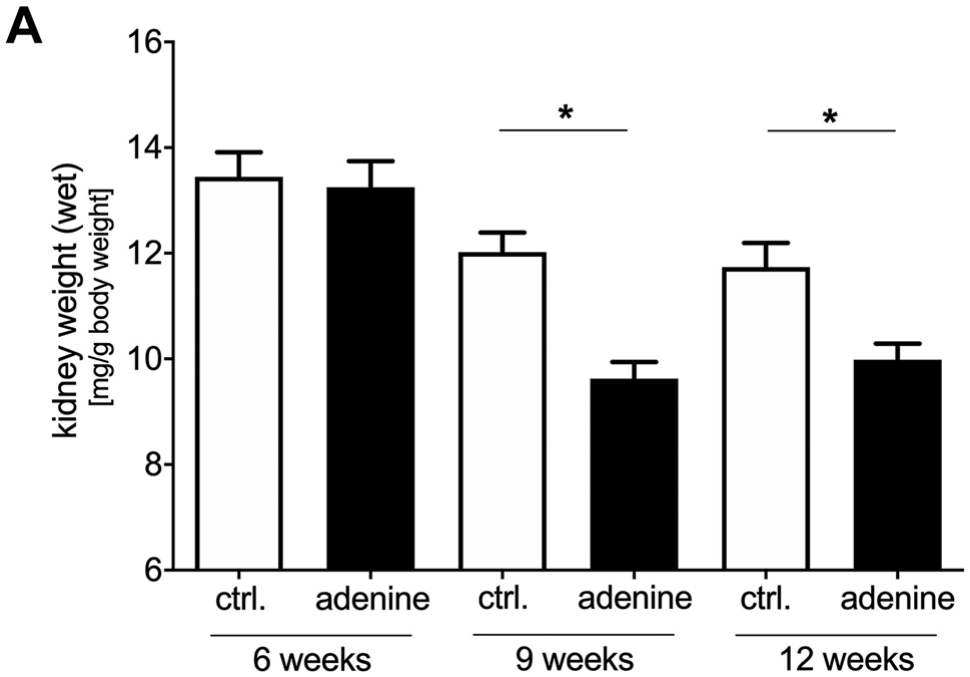
Kidney organ weight. Kidney organ weight (wet) normalized to the animal body weight of adenine-fed mice and control diet-fed mice. *p < 0.05 adenine vs. control

**Fig. 2:**
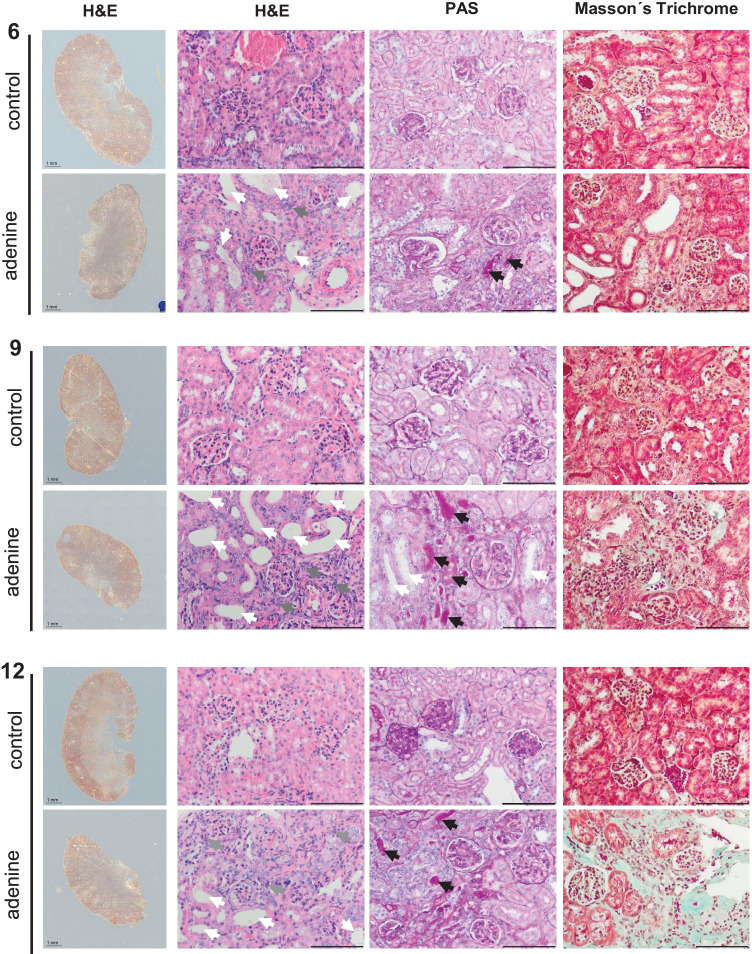
Histological renal changes. Hematoxylin and eosin (H&E), Perjodate Acid Schiff (PAS), and Masson’s trichrome staining of the kidney of control and adenine-fed mice over 6, 9, and 12 weeks (representative images). Arrows indicate dilated and atrophic tubules (white), tubulointerstitial infiltration with inflammatory cells (grey), and protein casts within the tubular lumen (black). Cyan-colored areas in Masson’s trichrome-stained images represent areas of renal fibrosis. Measuring bar represents 100 µm (except first row H&E: 1 mm)

Also, adenine-fed mice showed macroscopic and histological changes in the kidneys compared to controls. The kidney weight (normalized to the body weight) significantly decreased after 9 and 12 weeks (Fig. 1). In line, renal damage in adenine-treated animals as characterized by dilated and atrophic tubules, tubulointerstitial infiltration with inflammatory cells, and protein casts within the tubular lumen. In addition, a time-dependent increase in renal fibrosis was found in the adenine group compared to respective controls (Fig. 2).

### Vessel calcification in uremic mice

**Fig. 3:**
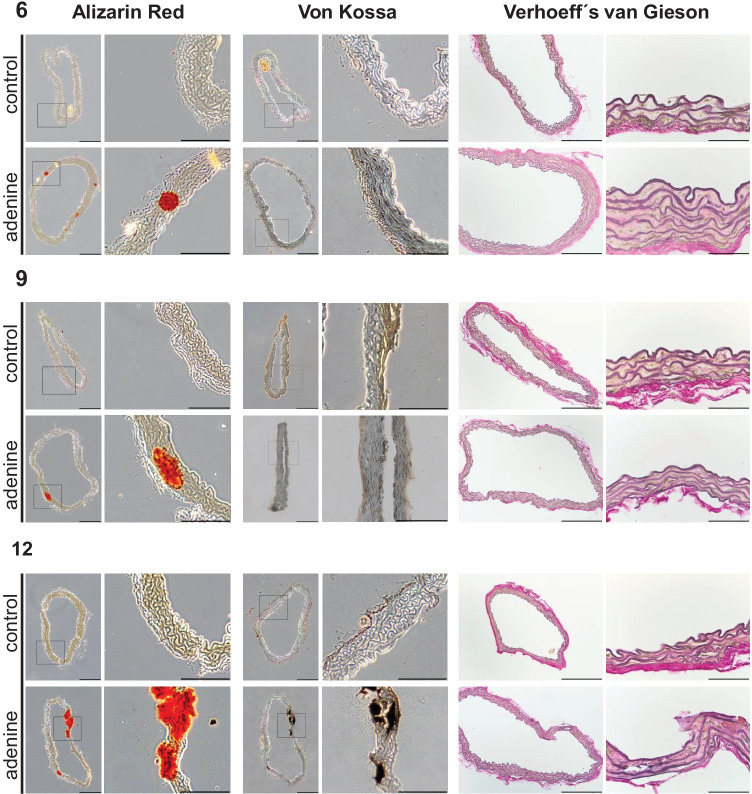
Calcification as shown by Alizarin Red staining and von Kossa staining as well as elastic tissue fibers Verhoeff’s van Gieson staining in aorta thoracalis at indicated time points (representative images). Measuring bar represents 100 µm for Alizarin Red and von Kossa images, 200 µm or 50 µm, respectively, for elastic tissue fibers Verhoeff´s van Gieson (EVG)

To test the effect of uremia induction on vessel calcification in mice, the amount of calcium phosphate in the thoracic vessel wall was determined by photometric quantification and visualized by histological staining. A significant and progressive calcification of the thoracic aorta, the abdominal aorta, and the renal artery appeared from the ninth week of treatment, which, as expected, was most pronounced in the thoracic aorta (Table 3). Histological staining with Alizarin Red and von Kossa confirmed the calcification of the vessel wall. In addition, there is an increase of elastin fibers’ disorganization in the adenine-fed group from week 6 to 12 (Fig. 3).

**Table 3:** Vessel mineralization in different vascular beds [mg calcium/µg tissue dry weight]

Vessel	Control (6 weeks)	Adenine (6 weeks)	Control (9 weeks)	Adenine (9 weeks)	Control (12 weeks)	Adenine (12 weeks)
Aorta thoracalis	65.5 ± 6.8	103.7 ± 14.3	49.0 ± 7.0	110.7 ± 17.9*	35.6 ± 6.1	75.1 ± 10.9*
Aorta abdominalis	41.2 ± 8.6	59.4 ± 14.8	64.8 ± 8.2	281.7. ± 53.2*	53.7 ± 10.5	233.7 ± 33.9*
Arteria renalis	62.1 ± 21.1	97.0 ± 16.7^a^	47.2 ± 10.1	158.8. ± 27.2*	39.3 ± 16.9^b^	**106.5 ± 24.2**^c^*
Arteria femoralis	37.5 ± 9.5^d^	43.0 ± 6.3	44.5 ± 15.7	87.1. ± 23.2	22.3 ± 2.5	79.4 ± 13.1*

If animal number is lower, not enough material could be selected: ^a^7 of 9, ^b^5 of 6, ^c^13 of 14, ^d^5 of 6. Mean ± SEM

^*^p < 0.05 adenine vs. respective control

**Fig. 4:**
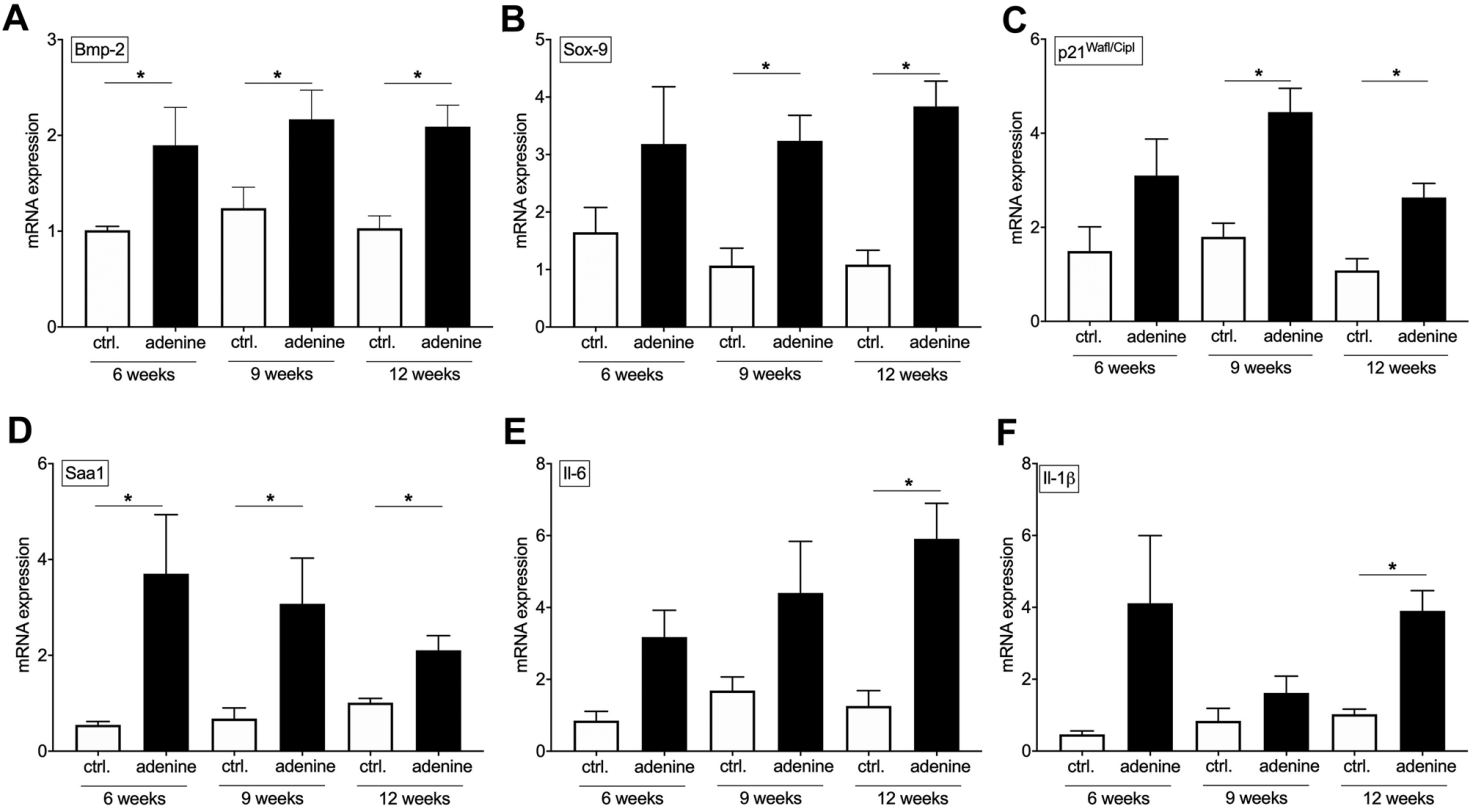
Aortic mRNA gene expression. Quantification of mRNA gene expression of the osteogenic markers: **a** bone morphogenetic protein-2 (Bmp-2) and **b** SRY box transcription factor 9 (Sox-9), the senescence marker **c** p21, as well as the pro-inflammatory markers **d** serum amyloid A (Saa), interleukin (Il) **e** Il-6 and **f** Il-1β in tissue of the *A. thoracalis* tissue in mice. Mean ± SEM, *p < 0.05 adenine vs. control

To evaluate the induction of transcriptional changes, the mRNA gene expression of established SASP parameters as well as of osteogenic, senescent, and pro-inflammatory markers was investigated [4]. Compared to the controls, the thoracic aorta of the CKD mice showed an increased expression of Bmp-2 and its transcription factor Sox-9 (Fig. 4A, B) as well as p21 (Fig. 4C). Previously, we identified Saa as a prominent pro-inflammatory uremic toxin (UT) in humans [23]. Il-1β and Il-6 are further UT modulating osteoblastic differentiation under uremic condition [9]. Therefore, the gene expression of these inflammatory SASP factors was examined and found to be significantly increased in CKD mice compared to control animals (Fig. 4D–F).

### Systemic SASP response

**Fig. 5:**
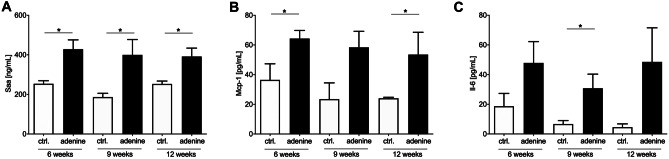
Systemic senescence associated secretory phenotype (SASP) response. Quantification of plasma cytokine levels as **a** serum amyloid A (Saa) by ELISA and **b**, **c** monocyte chemoattractant protein-1 (Mcp-1) and interleukin-6 (Il-6) by Luminex™ technique. Mean ± SEM, *p < 0.05 adenine vs. control

Because it is already known from human studies that inflammation and SASP are critically involved in initiation and progression of MAC [4, 5], the SASP profile under uremic condition in mice was quantified by the analysis of the systemic plasma concentration of 34 pro-inflammatory markers. Out of those, three increased under uremic conditions in adenine-fed mice: Saa, monocyte chemoattractant protein-1 (Mcp-1), and Il-6 (Fig. 5A–C). Further 18 investigated cytokines showed no significant regulation or only for one time point (Table S4) and 13 of the assessed cytokines were under detection limit (Table S5).

## Discussion

In the current study, we present a DBA2/N mice model for induction of renal failure via adenine-enriched diet. This model is suitable for studying uremic vascular calcification and arterial “inflamm-aging” as characterized by the induction of cellular senescence, pro-inflammatory SASP, and osteogenic differentiation. Although several studies have already investigated parts of this context [13–20], our experimental setting differs in several parameter to previously published protocols and therefore combines the advantages of non-surgical disease induction by likewise minimizing the disadvantages that were so far associated with feeding of an adenine-enriched diet: (1) no surgical intervention to induce CKD in mice; (2) the modified diet with 0.2% adenine, 1% phosphate, and 6% protein content is well tolerated by the mice and the weight loss is not associated with premature dead; and (3) to our knowledge, this is the first study demonstrating the adenine-induced time-dependent uremia progression with associated MAC and SASP in DBA2/N mice.

As it is already known for DBA2 mice that they suffer from an age-dependent soft tissue calcification and are more prone to develop MAC after CKD induction than other mice strains [11], this mice strain was used for the presented adenine-based CKD model. As female mice are more prone to calcification compared to male littermates [18], only female mice were selected for this study.

CKD induction in rodents can be induced either by surgical reduction of renal mass or by nephrotoxic adenine diet [11]. Nephrectomy-based CKD models are established for rats and mice, but the spread of renal function loss and associated MAC extent is high [11]. Induction by adenine leads to a reproducible CKD condition in rodents with moderate to severe MAC progression [11]. Drawbacks of the initial protocols using 0.75% adenine are the pronounced weight loss associated with high mortality rate and biological variability in the CKD degree [11]. A reduced mortality rate and reduced body weight loss could be achieved by reduction of the adenine content (0.25% in rats, 0.2% in mice) [11]. The adenine dose of the current study with 0.2% was sufficient to induce a stable uremic condition in all mice from 6 to 12 weeks without any impact on animal survival.

By variations of the diet components, the biological variations and weight loss can be also reduced. Some studies used vitamin D-enriched diet that on the one hand increases vessel calcification, but on the other hand further promotes weight loss [11]. A study by Price et al. showed in rats that reduction of the protein content in the adenine diet to 2.5% leads to exacerbated aortic calcification compared to higher protein content of 25% [24]. However, reduction of protein content leads also to reduction in food intake [25]. To counteract the expected weight loss associated with using the adenine-based protocol for renal insufficiency induction in mice, we chose a protein content of 6%. Although the protein content is reduced compared to the standard diet, we expected less reduction in food intake [12, 25]. In our study, the diet was tolerated by the animals and the weight loss was controllable so that no premature death occurred. The body weight loss upon adenine feeding was also seen in other studies with mice receiving a diet with similar adenine content [13].

The CKD induction is reflected by histological alterations of glomerular and tubular structures corresponded with an increase of blood parameters as Bun, Crea, and Pth. These results are in line with those of previous CKD studies in C57BL/6 mice [13], ldr knockout mice [26], and rats [12]. The laboratory chemistry reference areas were found to be different in various mice strains and varies also during the stages of animal growth [27]. A direct comparison of the blood parameters between different studies is also hampered by variations in ingredients of the experimental diet used; these ingredients do not only vary in concentration [11], but also in their source, which is relevant as, e.g., the intestinal resorption of phosphorus varies depending on the source. In our study, we used a diet containing 1% phosphate which did not result in elevated phosphate plasma concentration, while other protocols often used hyperphosphatemia inducing phosphate concentration (up to 2% phosphate) [14, 15, 28]. Tani et al. recently compared 0.8% phosphate vs. high phosphate concentration (1.8%) in C57/BL6 mice. After 12 weeks of treatment, they found higher phosphate plasma concentrations in both groups and detected a stable CKD and subsequent MAC [28]. In contrast, several studies in DBA2 mice showed no hyperphosphatemia with diet phosphate concentrations of 0.5% and 0.9%, respectively [18–20]. These findings are in line with our results.

Previously published results by Shanahan´s group showed the importance of premature vascular aging for MAC in children with advanced CKD in vivo [4]. Compared to controls, human vessels and VSMC showed properties of premature vascular aging and increased mineralization, which was partly due to activation of the pro-inflammatory SASP [4]. The SASP is established in different cell types in response to DNA damage and includes potent osteo-inductive and pro-inflammatory mediators like Bmp-2, Il-1β, and Il-6 [4, 29–32]. These data suggest that the paracrine secretion of pro-inflammatory and/or osteo-inductive factors by senescent VSMC may be an important driver of human uremic MAC. However, animal models with a comparable pathophysiology are required to investigate signal transduction pathways and treatment options. As already shown by Santana et al., adenine-induced CKD is associated with chronic inflammation in C57/BL6 mice [13]. However, the causal link to MAC remains unclear, as this study aimed to investigate the inflammatory-based renal insufficiency [13]. The investigated model presented here might bridge this gap. Thoracic aortas from uremic DBA2/N mice show properties of premature aging, osteogenic differentiation, and enhanced mineralization. Comparable to the situation in CKD children, the mouse aortas have enhanced expression of p21, Bmp-2, Sox-9, Saa1, Il-6, and Il-1β. In CKD children, the Il-6 plasma concentration was significantly elevated and showed a high correlation to the extent of coronary artery calcification [4]. Therefore, we investigated a pro-inflammatory multiplex panel in our mouse model. Interestingly, out of the 34 investigated cytokines, only the plasma concentration of Saa, Il-6, and Mcp-1 were increased. Possibly, a *vicious cycle* of inflammation during uremia leads to the predictive effect of cytokine release and cardiovascular death as shown for Saa and Il-6 [33–35]. In addition, studies have shown that the pathways of Il-1β and Il-6 are interconnected [36]. Recently, we could show that Il-6 is induced during calcification progression, but not directly induces mineralization, while Il-1β induces an osteogenic driven auto-loop in smooth muscle cells [37]. Il-1β and Il-6 are known middle-sized uremic toxins influencing osteogenic differentiation [9].

Limitations of the current protocol are (a) only female DBA2/N mice were investigated so that a transfer to other sex and strains is pending, (b) no blood and pulse pressure data were obtained, and (c) this study was designed as proof-of-experimental protocol study so that an interventional approach in the current model is also pending.

As some studies already exists for C57/BL6 mouse strains and CKD induction upon adenine diet [15, 16, 28], the current protocol should be transferable to other strains. A correlation between blood and pulse pressure changes with the extent of MAC was successfully shown in an adenine rat model [12]. Some animal treatment studies with therapeutic treatment are already available. The protective effect of tissue non-specific Alp inhibition in an adenine-based CKD mouse model was recently shown [28]. A further study has shown the potential of a treatment strategy by induction of an endogenous regulator against MAC, peroxisome proliferator-activated receptor-gamma coactivator-1 alpha in a CKD rat model [38]. Also, a magnesium-based phosphate binder as therapeutic option was tested in an adenine-based CKD rat model [39].

As up to now, specific treatment options in the clinical situation for reduction or prevention of disease progression are still missing, and of current research interest [1, 3], the described study protocol here might be beneficial in further studies.

We used an adenine-based diet to induce CKD in DBA2/N mice with subsequent chronic uremia resulting in MAC and associated SASP situation in the vessel wall of the mice. The described situation is comparable to the human CKD situation recently published [4] in several points: (1) premature aortic aging (e.g., p21), (2) osteogenic trans-differentiation (e.g., Bmp-2), and (3) and vessel mineralization.

Because CVD is a strong prognostic factor in CKD, understanding its pathogenesis as well as evaluating and testing of novel therapeutic drugs are urgently needed to provide new therapeutic concepts that help to reduce the high cardiovascular mortality of these patients, and therefore, suitable animal models are necessary.

## Supplementary Information

Below is the link to the electronic supplementary material.

Supplementary file1 (DOCX 462 KB)

## Data Availability

The datasets generated during the current study are not publicly available but are available from the corresponding author on reasonable request.
