# Supplementary material for: Uremic mouse model to study vascular calcification and “inflamm-aging”
Source: J Mol Med (Berl). 2022 Aug 2;100(9):1321–30. doi: 10.1007/s00109-022-02234-y (PMC9402761; doi:10.1007/s00109-022-02234-y)
Supplement: Supplementary file 1 — Supplementary file1 (DOCX 462 KB) [file 109_2022_2234_MOESM1_ESM.docx]

**Supplementary Material**

1. Animal study design


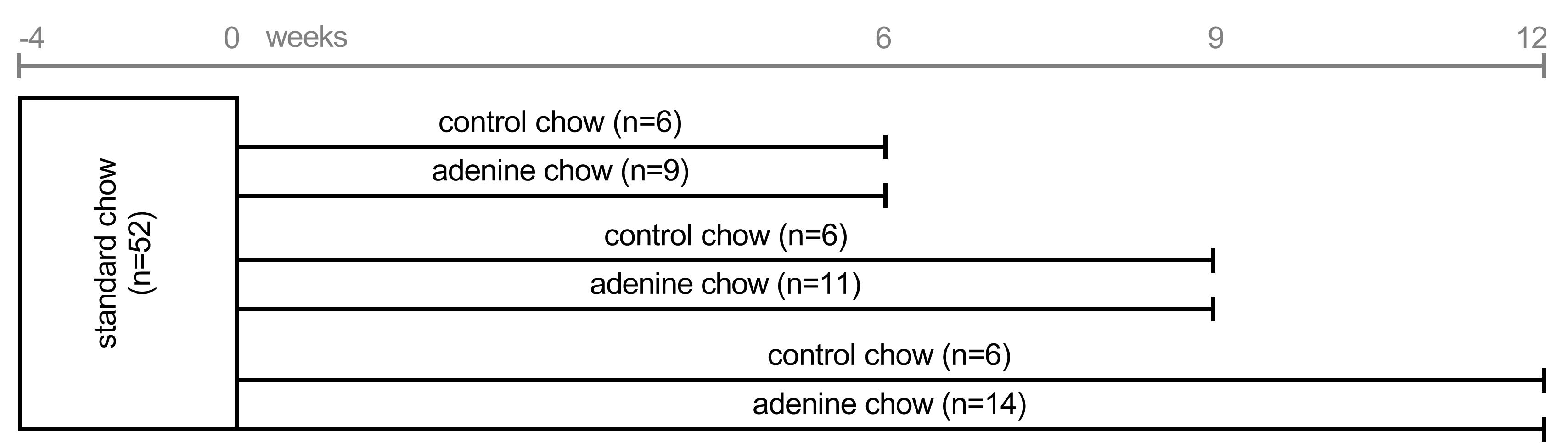


**Figure S1.** Experimental setting scheme.

Female DBA/2N were allowed to acclimatize for 4 weeks on standard chow prior the experiment started. The experimental approach was terminated at week 6, 9 and 12 with the declared animal numbers.

**Table S1.** Adenine-enriched diet ingredients according to manufacturer (Ssniff, Soest, Germany).

^1^Phosphorus added as calcium phosphate dibasic, potassium phosphate monobasic, sodium phosphate monobasic dihydrate, casein also serves as phosphorus source

| **Ingredients** | **%** |
| --- | --- |
| Casein | 6.800 |
| L-Cystine | 0.150 |
| Corn starch | 46.000 |
| Maltodextrin | 17.600 |
| Sucrose | 10.000 |
| Cellulose | 5.000 |
| Vitamin premix | 1.000 |
| Choline Cl | 0.250 |
| Soybean oil | 7.000 |
| Mineral premix^1^ | 6.000 |
| Adenine | 0.200 |

**Table S2.** Proximate contents of adenine-enriched diet to manufacturer (Ssniff, Soest, Germany).

^1^Phosphorus added as calcium phosphate dibasic, potassium phosphate monobasic, sodium phosphate monobasic dihydrate, casein also serves as phosphorus source

| **Proximate contents** | **%** |
| --- | --- |
| Crude protein | 6.000 |
| Crude fat | 7.100 |
| Crude fibre | 5.000 |
| Crude ash | 5.400 |
| Starch | 44.200 |
| Dextrin | 17.300 |
| Sugar | 11.300 |
| Calcium | 1.000 |
| Phosphorus^1^ | 1.000 |
| Sodium | 0.180 |
| Potassium | 0.4700 |
| Energy (MJ/kg)  Protein (kJ%)  Fat (kJ%)  Carbohydrates (kJ%) | 16  6  17  77 |


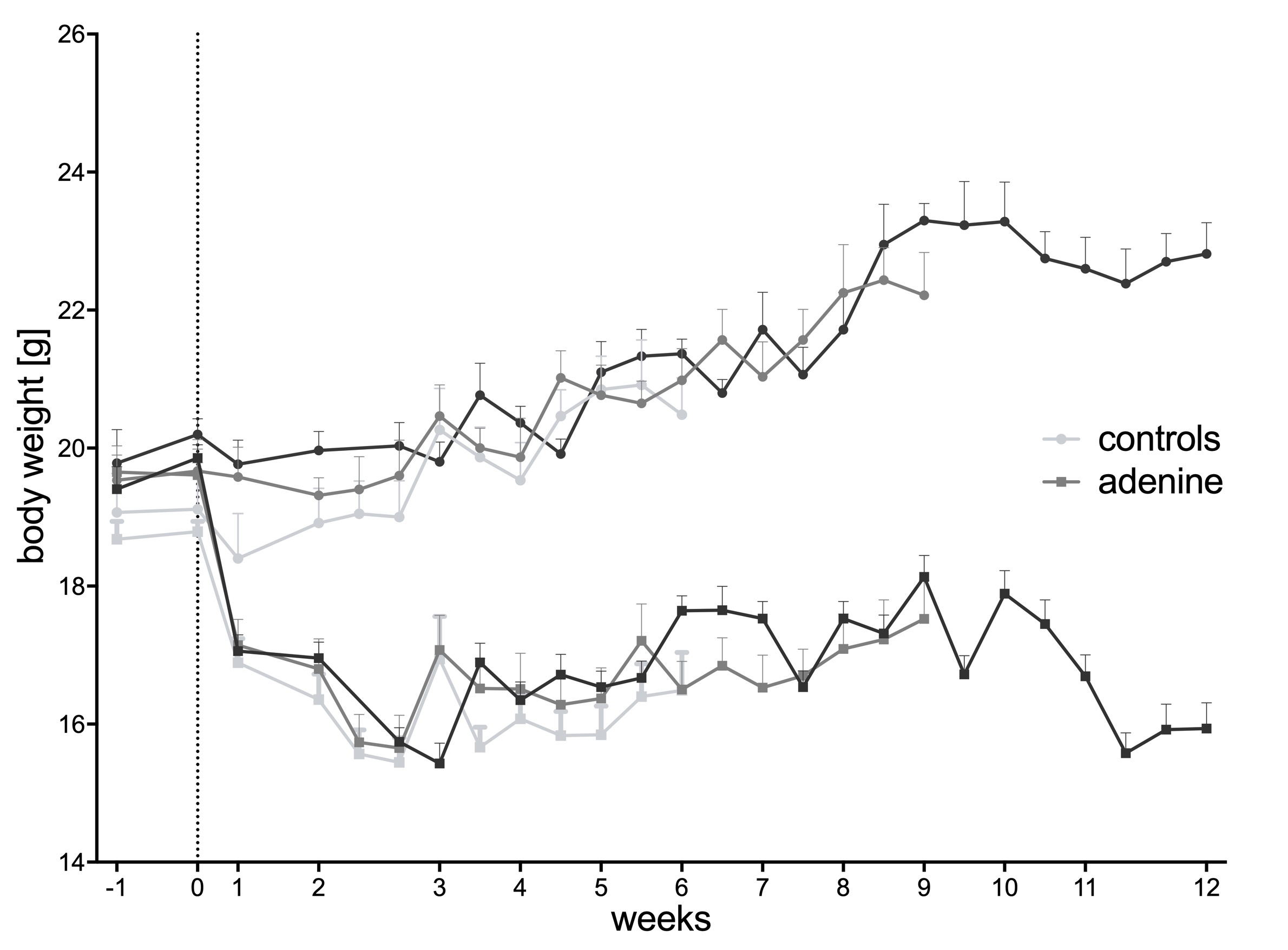


**Figure S2.** Weight differences over the observation period.

The female mice were weighed once per week for the first two weeks. Afterwards, the animal weight was determined two to three times per week. Control animal gain weight, whereas adenine groups lose weight in the first 2 weeks and afterwards barely gain weight.

2. mRNA gene expression

**Table S3.** Oligonucleotide sequences for quantitative real-time polymerase chain reaction

| **Gene** | **Oligo sequence 5´- 3´** | **Company** |
| --- | --- | --- |
| Sox-9 | Fwd: CAC ACG TCA AGC GAC CCA TGA A  Rev: TCT TCT CGC TCT CGT TCA GCA G | Origene |
| Bmp-2 | Fwd: AAC ACC GTG CGC AGC TTC CAT C  Rev: CGG AAG ATC TGG AGT TCT GCA G | Origene |
| Il-1β | Fwd: TGG ACC TTC CAG GAT GAG GAC A  Rev: GTT CAT CTC GGA GCC TGT AGT G | Biomol |
| Il-6 | Fwd: TAC CAC TTC ACA AGT CGG AGG C  Rev: CTG CAA GTG CAT CAT CGT TGT TC | Origene |
| Saa1 | Fwd: GGA GTC TGG GCT GCT GAG AAA A  Rev: TGT CTG TTG GCT TCC TGG TCA G | Origene |
| p21 | Fwd: TCG CTG TCT TGC ACT CTG GTG T  Rev: CCA ATC TGC GCT TGG AGT GAT AG | Origene |
| Actb | Fwd: AAGAGCTATGAGCTGCCTGA  Rev: TACGGATGTCAACGTCACAC | Biozol |
| Ppia | Fwd: AGCTCTGAGCACTGGAGAGA  Rev: GCCAGGACCTGTATGCTTTA | Biozol |
| RPl13a | Fwd: ATGACAAGAAAAAGCGGATG  Rev: CTTTTCTGCCTGTTTCCGTA | Biozol |

3. Cytokine plasma concentration

**Table S4.** Plasma cytokine level – additional

The n-number varies, because for some samples the values were below the detection limit.

| **Cytokine** | **Weeks** | **Mean ± SEM** control / adenine  **[pg/ml]** | **Detection Limit**  **MinDC + 2SD [pg/ml]** |
| --- | --- | --- | --- |
| TNFα | 6 | <OOR / 13.5 ± 1.7 | 3.4 |
|  | 9 | 6.3 ± 2.6 / 15.0 ± 2.3 |  |
|  | 12 | 13.1 ± 0 / 16.1 ± 3.0 |  |
| MIG | 6 | **286 ± 8 / 153 ± 33 *** | 3.2 |
|  | 9 | **215 ± 48 / 96 ± 13 *** |  |
|  | 12 | 122 ± 26 / 106 ± 16 |  |
| G-CSF | 6 | 687 ± 203 / 641 ± 91 | 2.7 |
|  | 9 | 354 ± 39 / 689 ± 217 |  |
|  | 12 | 253 ± 57 / 698 ± 242 |  |
| Eotaxin | 6 | 4,322 ± 831 / 4,243 ± 288 | 2.2 |
|  | 9 | **2,838 ± 242 / 3,889 ± 235 *** |  |
|  | 12 | 4,468 ± 1,246 / 4,169 ± 305 |  |
| IP-10 | 6 | 167 ± 12 / 166 ± 10 | 1.0 |
|  | 9 | 133 ± 15 / 154 ± 19 |  |
|  | 12 | 125 ± 9 / 142 ± 24 |  |
| KC | 6 | 361 ± 91 / 230 ± 67 | 4.5 |
|  | 9 | 235 ± 42 / 139 ± 38 |  |
|  | 12 | 347 ± 148 / 99 ± 18 |  |
| LIX | 6 | 4533 ± 602 / 3,042 ± 757 | 33.2 |
|  | 9 | 2,727 ± 2,009 / 4,417 ± 724 |  |
|  | 12 | 6,170 ± 1,738 / 4,661 ± 502 |  |
| M-CSF | 6 | 18 ± 4 / 14 ± 3 | 6.1 |
|  | 9 | < OOR / 6 ± 2 |  |
|  | 12 | 31 ± 26 / 8 ± 2 |  |
| MIP-1α | 6 | 75 ± 9 / 61 ± 7 | 12.5 |
|  | 9 | 56 ± 2 / 56 ± 9 |  |
|  | 12 | 60 ± 6 / 80 ± 29 |  |
| MIP-1β | 6 | 76 ± 13 / 69 ± 5 | 21.0 |
|  | 9 | 36 ± 2 / 44 ± 5 |  |
|  | 12 | 42 ± 9 / 38 ± 5 |  |
| RANTES | 6 | 47 ± 9 / 29 ± 3 | 5.2 |
|  | 9 | 30 ± 7 / 30 ± 2 |  |
|  | 12 | 37 ± 13 / 16 ± 3 |  |
| Il-1α | 6 | 345 ± 99 / 223 ± 59 | 18.8 |
|  | 9 | 167 ± 37 / 121 ± 41 |  |
|  | 12 | 409 ± 188 / 130 ± 43 |  |
| Il-4 | 6 | 0.8 ± 0.08 / 0.7 ± 0.03 | 0.5 |
|  | 9 | **0.4 ± 0.02 / 0.7 ± 0.12 *** |  |
|  | 12 | 0.6 ± 0.13 / 0.5 ± 0.11 |  |
| Il-5 | 6 | 18 ± 6 / 6 ± 4 | 1.6 |
|  | 9 | 4 ± 0 / 14 ± 9 |  |
|  | 12 | 7 ± 3 / 14± 6 |  |
| Il-9 | 6 | 248 ± 36 / 359 ± 171 | 28.1 |
|  | 9 | 100 ± 26 / 225 ± 69 |  |
|  | 12 | 101 ± 0 / 299 ± 70 |  |
| Il-13 | 6 | 301 ± 31 / 259 ± 16 | 20.0 |
|  | 9 | 224 ± 44 / 248 ± 14 |  |
|  | 12 | 236 ± 56 / 222 ± 19 |  |
| Il-15 | 6 | 113 ± 10 / 104 ± 10 | 9.5 |
|  | 9 | 39 ± 30 / 82 ± 14 |  |
|  | 12 | 53 ± 21 / 52 ± 9 |  |
| VEGF | 6 | 1.0 ± 0.2 / 1.3 ± 0.1 | 0.5 |
|  | 9 | 0.6 ± 0.2 / 1.2 ± 0.1 |  |
|  | 12 | 0.8 ± 0.1 / 0.8 ± 0.1 |  |

OOR: out of range

**Table S5.** Cytokines under detection limit

The minimum detectable concentration (MinDC) as analyzed by Millipore.

| **Cytokine** | **Detection Limit**  **MinDC + 2 SD [pg/ml]** |
| --- | --- |
| GM-CSF | 16.9 |
| INFƔ | 1.8 |
| Il-1β | 9.8 |
| Il-2 | 2.0 |
| Il-3 | 1.4 |
| Il-7 | 2.2 |
| Il-10 | 3.2 |
| Il-12 (p40) | 6.3 |
| Il-12 (p70) | 8.5 |
| Il-17 | 0.8 |
| LIF | 1.3 |
| MIP-2 | 46.5 |
